# A glance at the gut microbiota and the functional roles of the microbes based on marmot fecal samples

**DOI:** 10.3389/fmicb.2023.1035944

**Published:** 2023-04-14

**Authors:** Chuizhe Chen, Shu Chen, Bo Wang

**Affiliations:** ^1^Department of Pathology, Hainan General Hospital, Hainan Affiliated Hospital of Hainan Medical University, Haikou, China; ^2^Key Laboratory of Tropical Translational Medicine of Ministry of Education, NHC Key Laboratory of Tropical Disease Control, School of Tropical Medicine and the Second Affiliated Hospital, Hainan Medical University, Haikou, China; ^3^Medical Laboratory Center, Hainan General Hospital, Hainan Affiliated Hospital of Hainan Medical University, Haikou, China

**Keywords:** marmot, gut microbiota, metabolism, metagenomic, resistance

## Abstract

Research on the gut microbiota, which involves a large and complex microbial community, is an important part of infectious disease control. In China, few studies have been reported on the diversity of the gut microbiota of wild marmots. To obtain full details of the gut microbiota, including bacteria, fungi, viruses and archaea, in wild marmots, we have sequenced metagenomes from five sample-sites feces on the Hulun Buir Grassland in Inner Mongolia, China. We have created a comprehensive database of bacterial, fungal, viral, and archaeal genomes and aligned metagenomic sequences (determined based on marmot fecal samples) against the database. We delineated the detailed and distinct gut microbiota structures of marmots. A total of 5,891 bacteria, 233 viruses, 236 fungi, and 217 archaea were found. The dominant bacterial phyla were Firmicutes, Proteobacteria, Bacteroidetes, and Actinomycetes. The viral families were Myoviridae, Siphoviridae, Phycodnaviridae, Herpesviridae and Podoviridae. The dominant fungi phyla were Ascomycota, Basidiomycota, and Blastocladiomycota. The dominant archaea were Biobacteria, Omoarchaea, Nanoarchaea, and Microbacteria. Furthermore, the gut microbiota was affected by host species and environment, and environment was the most important factor. There were 36,989 glycoside hydrolase genes in the microbiota, with 365 genes homologous to genes encoding β-glucosidase, cellulase, and cellulose β-1,4-cellobiosidase. Additionally, antibiotic resistance genes such as macB, bcrA, and msbA were abundant. To sum up, the gut microbiota of marmot had population diversity and functional diversity, which provides a basis for further research on the regulatory effects of the gut microbiota on the host. In addition, metagenomics revealed that the gut microbiota of marmots can degrade cellulose and hemicellulose.

## Introduction

Trillions of microbes inhabit the guts of animals, forming a dynamic ecological community within the gut, which is termed the “gut microbiota” (Ley et al., 2008; Lloyd-Price et al., 2016). The gut microbiota can affect the physiological and pathological states of the host by modulating the host’s metabolism and immune system (Tremaroli and Backhed, 2012; Maurice et al., 2013; Qin et al., 2014; Zhernakova et al., 2016). Bacteria are the most abundant microbes in the gut of mammals and they are therefore more significant than other members of the gut microbiota (Human Microbiome Project, 2012). Fungi can affect cellulose degradation and fermentation in herbivores (Huffnagle and Noverr, 2013; Sokol et al., 2017). Bacteriophages are abundant members of the gut microbiota and their genetic diversity is higher than that of bacteria or hosts (Reyes et al., 2013). They can contribute to gut inflammation and bacterial dysbiosis (Norman et al., 2015; Lusiak-Szelachowska et al., 2017). Archaea are key components of complex microbial communities in the environment and an important part of the animal gut microbiota (Moissl-Eichinger et al., 2018).

Marmots, which have been known since antiquity and found in the fossil record, were recognized by Thorington and Hoffman (2005) and placed in the family Sciuridae and genus marmot (Arnold, 2019). They are a ground-dwelling rodent mammal, mainly consisting of gray marmots, Himalayan marmots, and North American marmots (Cardini, 2003). They tend to live in mountainous and alpine meadow areas such as the northern of China, Eurasian Steppe, and in Europe and northwestern Asia. They feed on grass shoots, roots, stems, and leaves, and hibernate in winter (Armitage et al., 2003; Yan et al., 2015). The gut microbiota greatly affects the digestibility of cellulose and hemicellulose (Zhu et al., 2011; Xue et al., 2015). Cellulase (EC 3.2.1.4), β-glucosidase (EC 3.2.1.21) and cellulose 1,4-β-cellobiosidase (EC 3.2.1.91) are essential enzymes for digestion of cellulose and hemicellulose (Kubicek et al., 2009; Yang et al., 2018), and they can be produced by the gut microbiota.

Inner Mongolia, China, is the habitat of a variety of rare and protected wild animals such as marmots, sika deer, sables, and other unique species. The Hulun Buir grassland of Inner Mongolia is one of the four major marmot foci in China. Epidemiological studies have shown that the human plague strains were transmitted from Tibetan sheep, while the Tibetan sheep plague strains originated from marmots (Dai et al., 2018). Infected animal tissues can retain pathogens, and humans can be infected when hunting, trapping, or handling the infected animal tissues (He et al., 2021). Marmots (scientific name is Marmota bobak) of the Hulun Buir Plateau may be carrying various pathogenic microbes. However, research on marmots is limited to animal plague surveillance, and there is little information on the diversity of the gut microbiota (Kehrmann et al., 2020). Moreover, marmots mainly eat various types of grasses, stems, and leaves, and studying the gut microbiota may indicate whether marmots have gut microbiota that is specially adapted to a fiber-rich diet. Finally, the habitat of marmots is near to human living environments and farms, and the gut microbiota may reveal details about the local antibiotic use. In-depth research on the gut microbiota, which involves a large and complex microbial community rich in bacteria and viruses, is an important part of infectious disease control.

In this study, we collected the feces of marmots in the Hulun Buir region of Inner Mongolia to investigate the gut microbiota. We performed a systematic metagenomic analysis of the gut microbiota of marmots. We explored the marmot gut microbiota composition, diversity, and microbial roles. Moreover, we provide a description of the bacteria and fungi with cellulose degradation genes, glycoside hydrolase (GH) genes, and antibiotic resistance genes (ARGs) to evaluate the microbiota’s capacity for the degradation of cellulose and hemicellulose, starch hydrolysis, and antibiotic resistance. These results are an important step toward a better understanding of environment–diet–microbe–host interactions. In addition, we provide basic data for the future development of new ideas for the prevention and control of infectious diseases such as plague.

## Materials and methods

### Sample collection

We selected five locations in the Hulun Buir Plateau in Inner Mongolia, China. Three fecal samples were randomly taken from each location, with a total of 15 samples. The three fecal samples from each location were mixed into one group, giving a total of five groups. The fecal samples were collected and then immediately stored at −20 °C. After transport on dry ice, the fecal samples were stored at −80 °C.

### DNA extraction and sequencing

DNA in the fecal samples was extracted using The E.Z.N.A.® Stool DNA Kit (D4015-02, Omega, Inc., United States) according to the manufacturer’s instructions. DNA libraries were constructed using a TruSeq Nano DNA LT Library Preparation Kit (FC-121-4,001, USA). Metagenomic sequencing was performed on an Illumina HiSeq4000 platform in the PE150 mode.

### Annotation of metagenomes

We obtained metagenomic sequencing reads from the marmot fecal samples. First, we used internal scripts to remove the adapters, duplicates, and low-quality reads to obtain relatively high-quality data. Second, reads with length < 90 bp, truncated N content >5%, or host sequence contamination were removed. The remaining cleaned reads were used for microbial analysis.

The NR database is a non-redundant protein database from the National Center for Biotechnology Information (NCBI). It contains non-redundant sequences translated from GenBank nucleic acid sequences, along with non-redundant sequences from other protein databases, including RefSeq, PDB, SwissProt, PIR, and PRF. Sequences belonging to bacteria, archaea, viruses, and fungi were extracted from the NR database (version 2016.07.12). There were 52,375,954 sequences, which constituted the NR_meta library.

We then used DIAMOND software to align unigenes with the NR_meta library (blastp, evalue ≤1e-5). The alignment results of each unigene were compared, and the alignment results with evalue ≤minimum evalue*10 were selected for species classification. Using the NCBI species classification system and the lowest common ancestor (LCA) algorithm, we then obtained annotation information for each sequence at the kingdom, phylum, class, order, family, genus, species levels.

### Gene function annotation

First, the non-redundant reads in each sample were subjected to genome assembly using IDBA-UD software.^1^ Second, the coding regions (CDSs) were predicted and compared with BLASTP databases using MetaGeneMark, based on all contigs with length > 50 bp. CDSs length < 100 nt were removed. CD-HIT software was then used to compare non-redundant protein sequences to the NR database and identify the sequences with 95% similarity (Li and Godzik, 2006). Next, we clustered the sequences with a coverage of 90%, and then selected the longest sequences as the representative sequences. Thereafter, we used the Comprehensive Antibiotic Resistance Database (CARD), Gene Ontology (GO) database, Kyoto Encyclopedia of Genes and Genomes (KEGG) database (Kanehisa et al., 2017), and Carbohydrate-Active EnZymes (CAZy) database (Cantarel et al., 2009) to functionally annotated of these genes.

### Data analysis

We used GraPhlAn to generate taxonomic and phylogenetic trees with circular representations (Asnicar et al., 2015; Zhernakova et al., 2016). The linear discriminant analysis effect size (LEfSe) was displayed using Galaxy (LDA scores >2, *p* < 0.05)^2^ (Segata et al., 2011). An overview of the KEGG metabolic pathways was visualized using iPath 3.0 (Yamada et al., 2011). Circos plot of ARGs and AROs was displayed using Circos.^3^ Other graphs were constructed using GraphPad Prism 7.

## Results

### Metagenomic DNA sequencing

The gut microbiota was analyzed using paired-end metagenomic high-throughput sequencing on a HiSeq4000 platform, which generated a mean of 6.82 Gb (about 22.7 M reads) per sample (Supplementary Table S1). We predicted and statistically analyzed the abundances of microbial taxa from four different domains (bacteria, fungi, viruses, and archaea). The majority of reads were from bacteria (80.91%), 0.13% were from archaea, 0.05% were from viruses, 0.08% were from eukaryotes, and 18.83% were unclassified. At the phylum level, Bacteroidetes, Firmicutes, Proteobacteria, and Actinobacteria dominated (Supplementary Figure S1A). At the genus level, Clostridium, Firmicutes_noname, Ruminococcus, Lachnospiraceae_noname and Bacteroides dominated (Supplementary Figure S1B).

### Diversity of bacteria

**Figure 1:**
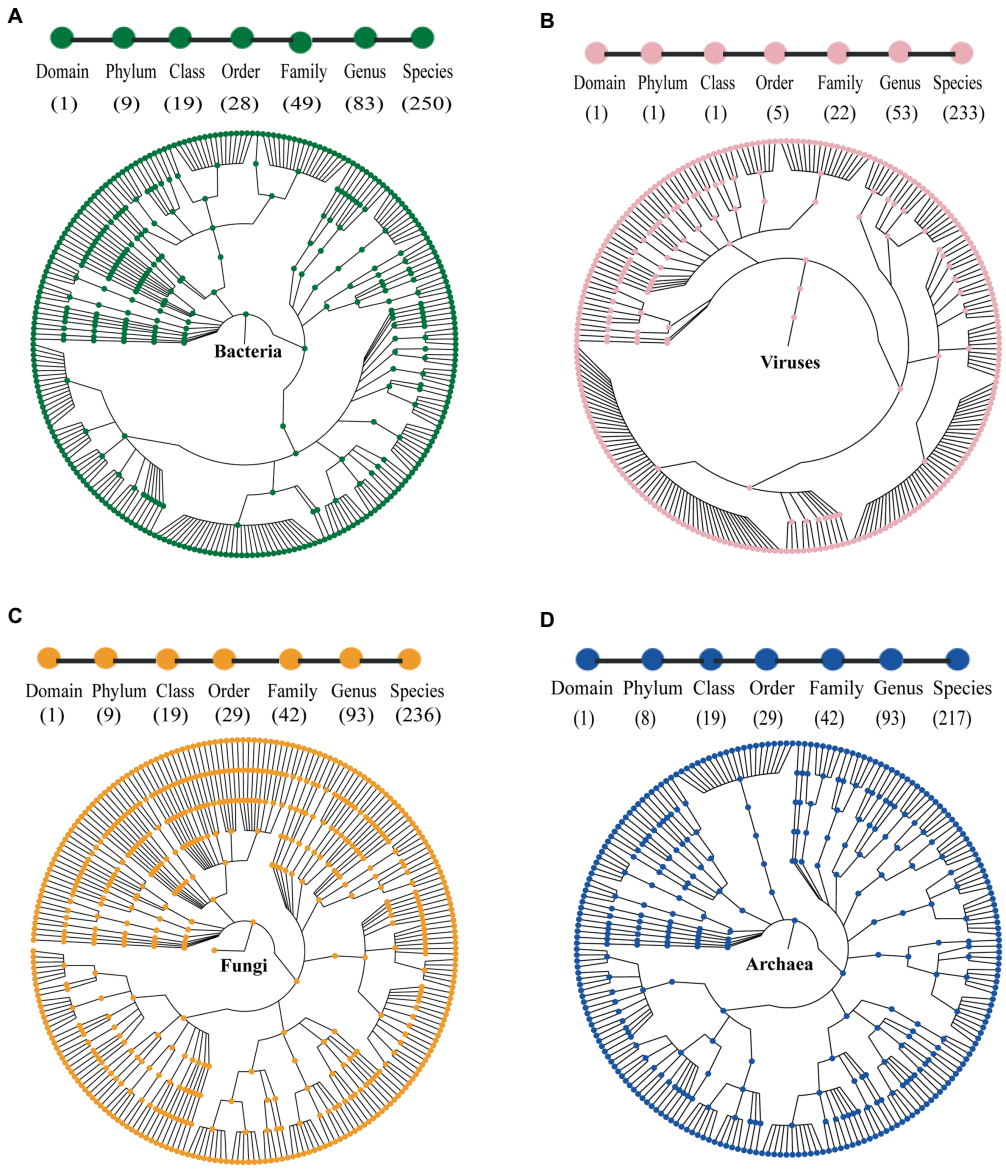
Taxonomic trees of **(A)** bacteria; **(B)** viruses; **(C)** fungi; and **(D)** archaea in the gut of marmots, predicted by MGS (metagenomics sequencing). From the inner to outer circles, the taxonomic levels range from kingdom to species. Numbers in parentheses indicate the total number of unique taxonomies detected at each level.

**Figure 2:**
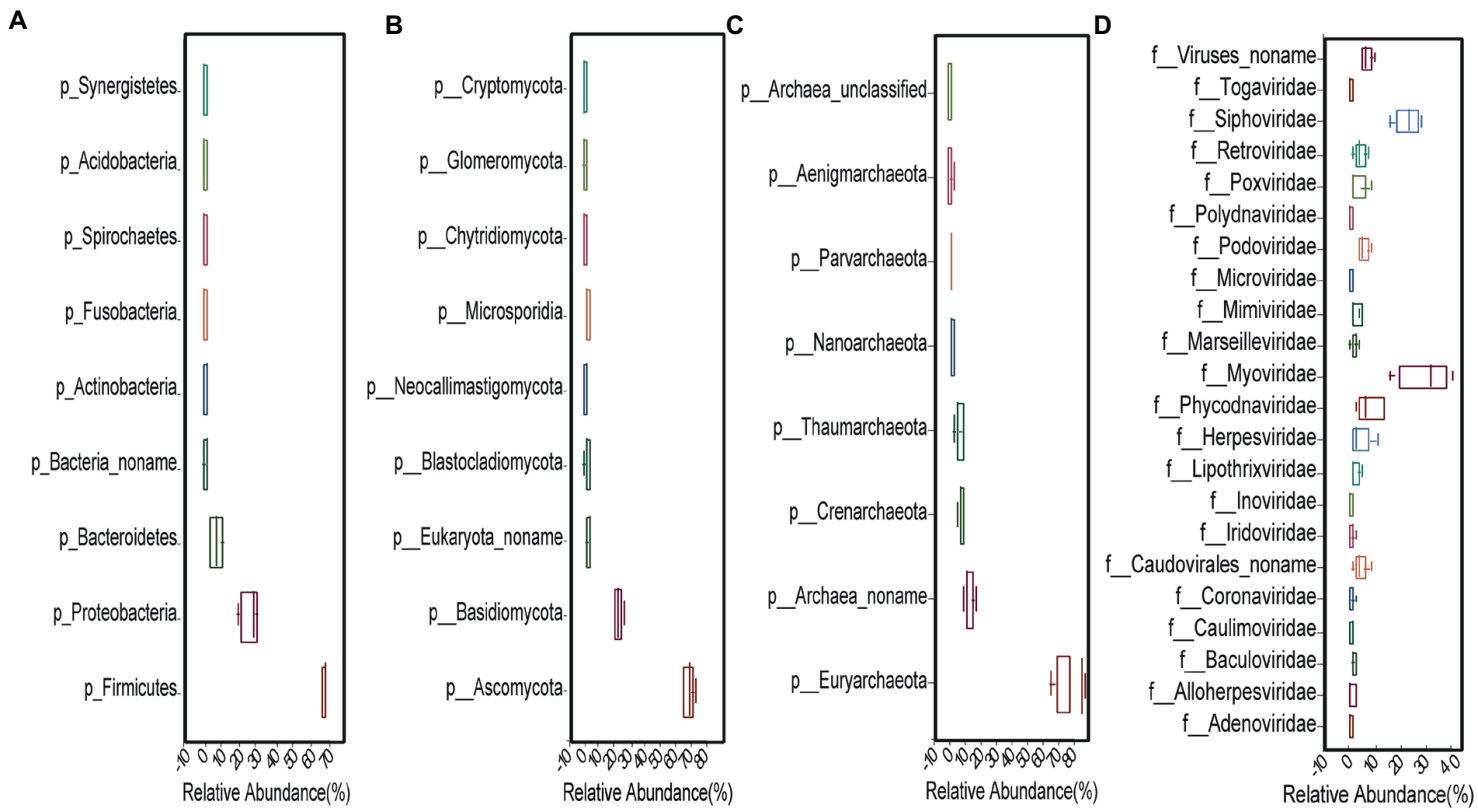
Relative abundances of **(A)** bacteria, **(B)** fungi, and **(C)** archaea at phyla level and **(D)** viruses at family level.

**Figure 3:**
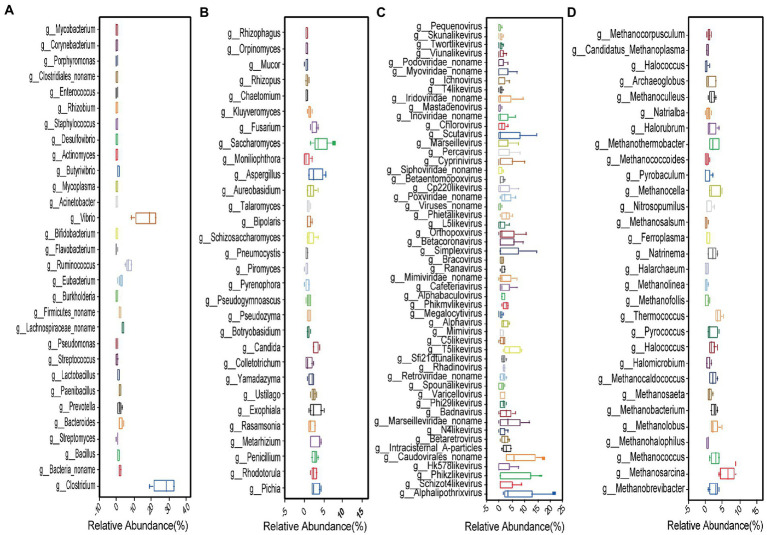
Top 30 most abundant **(A)** bacteria; **(B)** fungi; **(C)** viruses; and **(D)** archaea genera.

Compared to fungi, viruses, and archaea, bacteria were highly abundant. We found 5,891 bacterial species and selected the top 250 species based on relative abundance. There were 9 classified phyla, 19 classified classes, 28 classified orders, 49 classified families, and 83 classified genera (Figure 1A). The two most abundant phyla were Firmicutes (65.94%) and Proteobacteria (25.52%), followed by Bacteroidetes (6.18%), Actinobacteria (0.76%), Fusobacteria (0.26%), Spirochaetes (0.25%), and others (1.09%) (Figure 2A). The 30 most abundant genera are shown in Figure 3A and the top 10 are listed in Supplementary Table S2. The dominant genera were Clostridium, Vibrio, and Ruminococcus. The top 50 species are shown in Supplementary Figure S2A and the top 10 are listed in Supplementary Table S3.

### Diversity of fungi

We found 236 fungal species, involving 9 classified phyla, 19 classified classes, 29 classified orders, 42 classified families, and 93 classified genera (Figure 1C). The dominant phyla were Ascomycota (68.63%) and Basidiomycota (22.46%), followed by Blastocladiomycota (1.9%), Microsporidia (1.51%), Neocallimastigomycota (1.32%) and others (0.98%) (Figure 2B). The top 30 genera are shown in Figure 3B and the top 10 are listed in Supplementary Table S2. The top 50 species are shown in Supplementary Figure S2C and the top 10 species are listed in Supplementary Table S3.

### Diversity of viruses

We found 233 viral species, involving 5 classified orders, 22 classified families, and 53 classified genera (Figure 1B). The seven most abundant classified families were Myoviridae (28.9%), Siphoviridae (23.09%), Phycodnaviridae (7.85%), Podoviridae (5.32%), Herpesviridae (4.48%), Caudovirales (4.39%), and Retroviridae (4.26%) (Figure 2D). All 53 genera are shown in Figure 3C and the top 10 are listed in Supplementary Table S2. The top 50 species (25 species had >1% abundance) are shown in Supplementary Figure S2B and the top 10 are listed in Supplementary Table S3.

### Diversity of archaea

We found 217 archaeal species, involving 7 classified and 1 unclassified phyla, 16 classified and 3unclassified classes, 26 classified and 3 unclassified orders, 39 classified and 3 unclassified families, and 90 classified and 3 unclassified genera (Figure 1D). The dominant phyla were Euryarchaeota (74.52%), Crenarchaeota (6.39%), and Thaumarchaeota (4.95%) (Figure 2C). The top 30 genera are shown in Figure 3D and the top 10 are listed in Supplementary Table S2. The top 50 species are shown in Supplementary Figure S2D and the top 10 are listed in Supplementary Table S3.

### Host species and environment affected gut microbiota diversity in marmots

To determine whether the gut microbiota was affected by host species and the environment, we analyzed the gut microbiota structure of marmots in Hulun Buir grassland and that of other herbivores. Gut microbiota data of wild herbivores in Austria, i.e., Alpine marmots (scientific name is Marmota marmota; sample accession no. ERS2859683) and European rabbits (scientific name is Oryctolagus cuniculus; sample accession no. ERS2859628), were obtained from Animal Microbiome Database (AMDB^4^).

**Figure 4:**
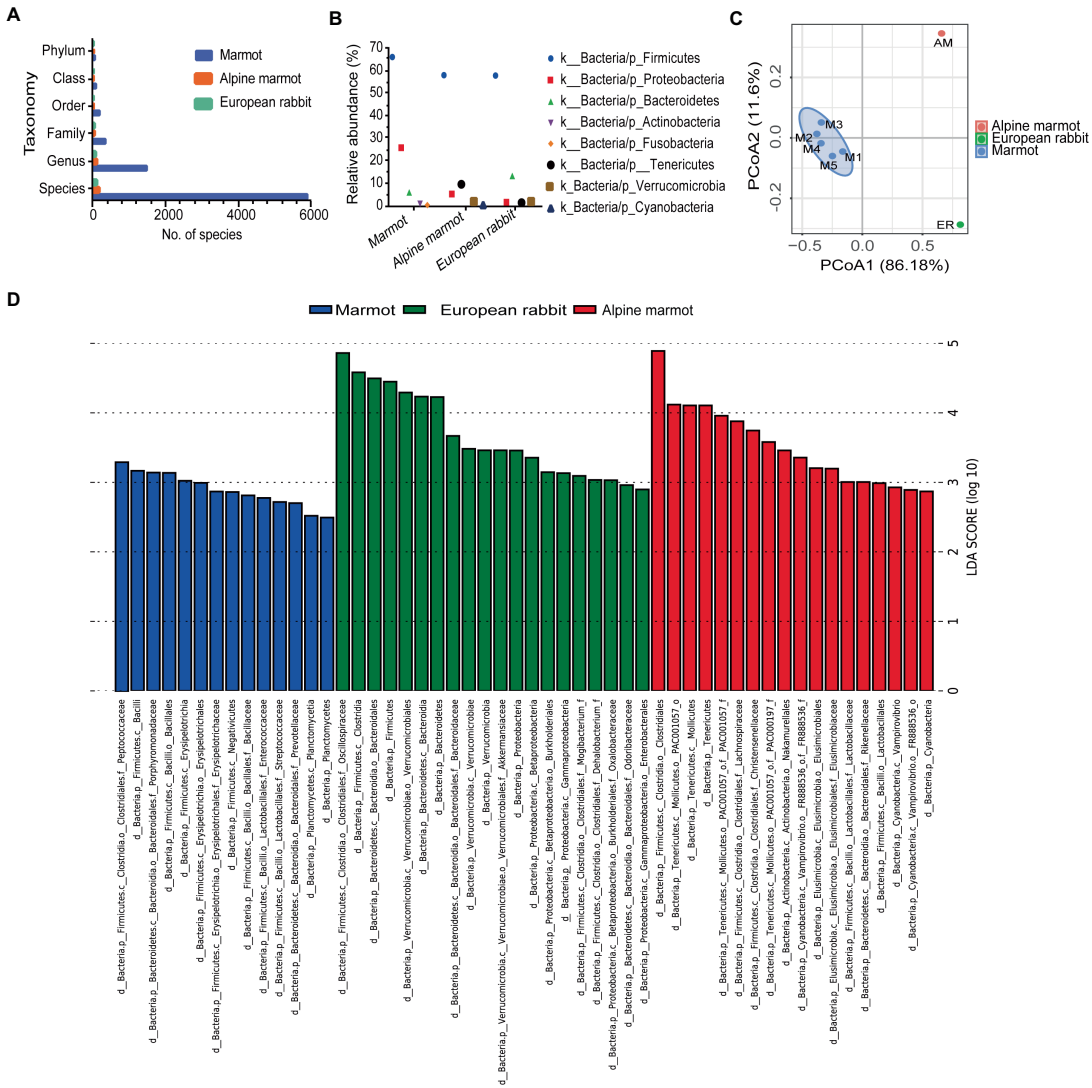
Gut microbiota of marmots and other wild herbivores. **(A)** The bar plots summarize the number of species per host. **(B)** The top five most abundant phyla for per host are displayed. **(C)** PCoA analysis of species. Each point in the figure represents a sample, and samples of the same group are represented by the same color. **(D)** LEfSe identification of several microbial families with significant differences (LDA > 2, *p* < 0.05).

In these animals, the most dominant kingdom in the gut microbiota was bacteria. For each bacterial taxa, the diversity was higher in marmots in Hulun Buir grassland than Alpine marmots and European rabbits (Figure 4A). The top 5 most abundant phyla for each of the three herbivores are displayed in Figure 4B. Firmicutes and Proteobacteria were present in all three herbivores, and Firmicutes was the most abundant phylum (Figure 4B). Based on the relative abundances of species, we conducted a PCoA analysis of differences in gut microbiota between the Marmots, Alpine marmots and European rabbits (Figure 4C). We found that the microbial species composition of the five samples in marmots were relatively similar. However, the microbial species composition of different species was different. Furthermore, we found that the marmots, Alpine marmots, and European rabbits harbored 52 significantly different bacteria taxa using LEfSe (LDA Effect Size) analysis (Figure 4D, LDA score > 2, *p* < 0.05). Firmicutes, Bacteroidetes and Planctomycetes were markedly more abundant in the gut microbiota of marmots; Verrucomicrobia and proteobacteria were markedly more abundant taxa in the gut microbiota of European rabbits; Tenericutes, Actinobacteria and Cyanobacteria were markedly more abundant in the gut microbiota of Alpine marmots (Figure 4D). The significant difference between marmots, Alpine marmots and European rabbits suggested that the function of the gut microbiota was closely associated with the host species and diet of the host. In addition, we measured the effects of host species and environment variation using Bray–Curtis dissimilarities (BC_ij_) in order to explore the factors that shape the gut microbiota. The BC_ij_ of marmots–Alpine marmots, marmots–European rabbits, and Alpine marmots–European rabbits were 0.93, 0.96 and 0.28, respectively. These results indicate that host species and environment affected the gut microbiota of marmots, and environment was the most important factor.

### Functional annotation and classification

**Figure 5:**
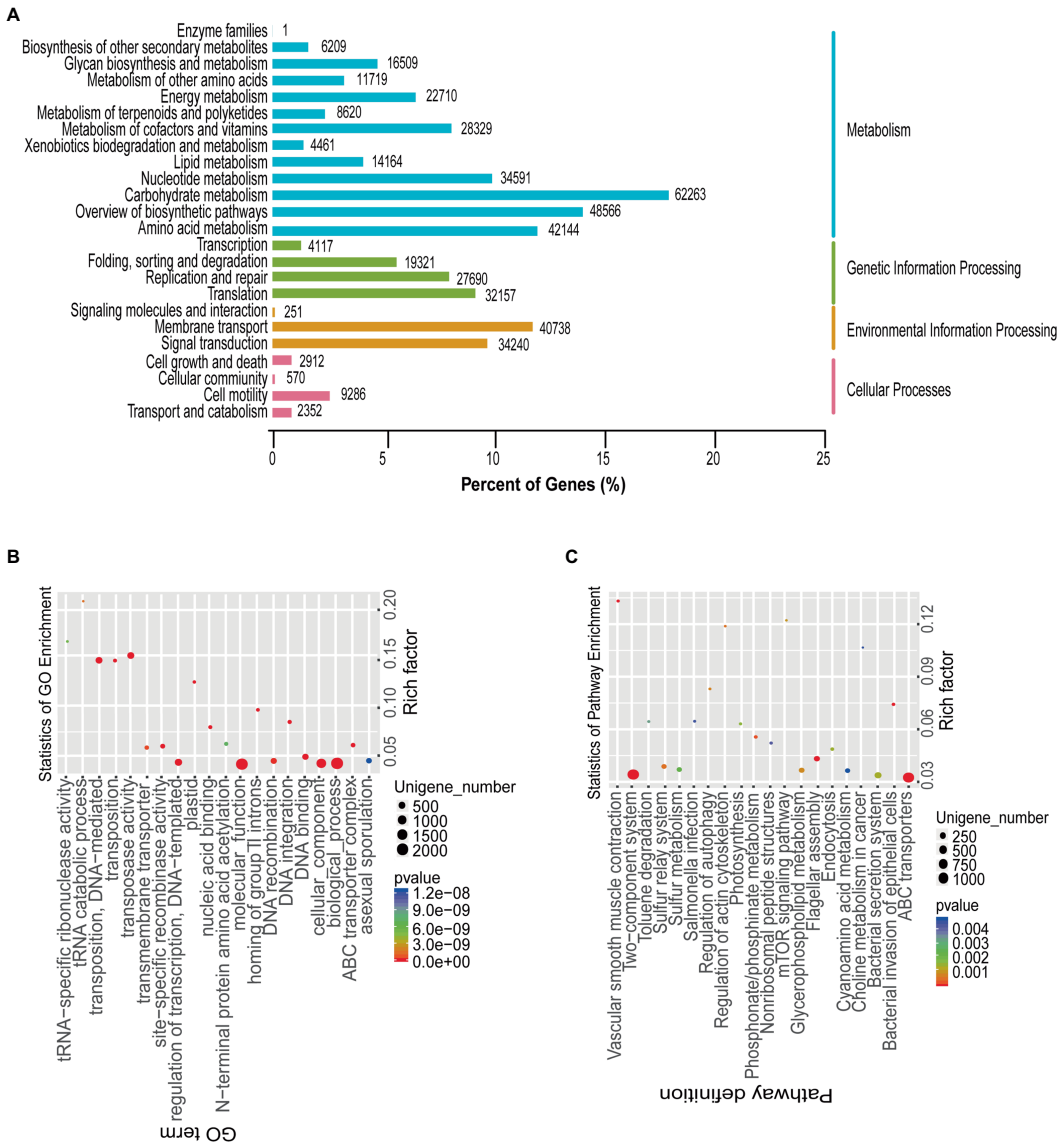
Functional annotation of gut microbiota. **(A)** KEGG pathway classification. The left and right vertical axes show the KEGG level 2 and KEGG level 1 classification information of KEGG PATHWAY, and the horizontal axis shows the percentage of annotated unigenes. **(B)** Differential gene enrichment analyzes. Scatter diagram of GO analysis results, using ggplot2 in R. Rich factor: number of differential genes with the GO term/total number of genes with the GO term. The larger the Rich factor, the higher the enrichment of the GO term. **(C)** Differential gene enrichment analyzes. Scatter diagram of KEGG analysis results, using ggplot2 in R. Rich factor: number of differential genes with the KEGG pathway/total number of genes with the KEGG pathway. The larger the Rich factor, the higher the enrichment of the KEGG pathway.

To understand the gene functions of the marmot gut microbes, we performed functional annotation of the sequencing results using KEGG and GO analyzes. According to the KEGG annotation summary table, combined with the hierarchical structure of the KEGG PATHWAY database, the KEGG PATHWAY level1 and 2 are shown in Figure 5A. Regarding the KEGG level1 results, the most dominant was Metabolism (59.82%), followed by Genetic Information Processing (16.59%), Environmental Information Processing (14.99%), and Cellular Processes (3.01%) (Supplementary Table S4). In addition, we used the GO database to perform GO functional annotations of the five sample groups. The GO annotations in order of dominance were Molecular Function (26.0%), Biological Process (24.5%), and Cellular Component (12.5%) (Supplementary Table S4).

Due to the diversity of individuals, analysis of the five metagenomes revealed that 39,434 genes were significantly differentially co-expressed. GO analysis of the differential genes revealed the functional diversity of these differential genes, involving tRNA−specific ribonuclease activity, tRNA catabolic process, transposase activity, regulation of transcription, ATP − binding cassette (ABC) transporter complex, and other (Figure 5B). KEGG analysis of the differential genes also revealed functional diversity, involving Cyanoamino acid metabolism, Salmonella infection, ABC transporters, Choline metabolism in cancer, Nonribosomal peptide structures, Regulation of actin cytoskeleton, mTOR signaling pathway, and other (Figure 5C).

### Antibiotic resistance genes, AROs, and gut microbiota

**Figure 6:**
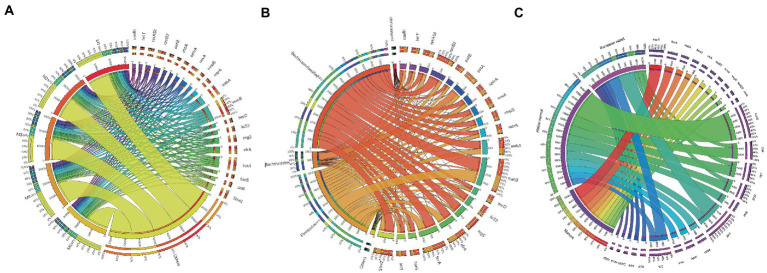
Antibiotic resistant genes (ARGs) types and their abundances. **(A)** Circos plot of relative abundances of ARGs. Right side shows antibiotic information and left side shows sample information. Outer circle indicates distribution of unique genes and inner circle shows different samples or antibiotics. **(B)** Distribution of antibiotic resistance ontologies (AROs) by phyla. **(C)** Circos plot of relative abundances of ARGs in marmot, Alpine marmot, and European rabbit.

The use of high concentrations of antibiotics in the agriculture and livestock industries has led to strong selection pressure, which promotes the exchange of antibiotic resistance genes (ARGs) between pathogens and gut microbiota. To understand the ARGs and the microbial sources of antibiotic resistance ontologies (AROs) in the gut microbiota of marmots, with the history of antibiotic use of agriculture and animal husbandry in local we used the Comprehensive Antibiotic Resistance Database (CARD) to screen for antibiotic resistance factors among the unigenes based on the metagenome data. CARD was used to annotate 29,416 genes, and 471 AROs were identified. The top 20 most abundant AROs in each sample are displayed in Figure 6. We identified ARGs in all samples and found that macB had the highest abundance, followed by bcrA, msbA, rpoB2, efrA, and stre2 (Figure 6A). A range of ARG categories were identified, including resistance to macrolides, peptides, nitroimidazole, aminocoumarin, fluoroquinolone, tetracycline, mupirocin, lincosamide, oxazolidinone, phenicol, pleuromutilin, streptogramin, acridine dye, glycopeptide, and aminoglycoside. These ARG categories involve some antibiotics that have been used for the prevention and treatment in the local agriculture and animal husbandry industries in Hulun Buir City in Inner Mongolia, China. In addition, we analyzed the microbial sources of AROs and found that they aggregated together; Except for Bacteria_noname and Bacteria_unclassified, approximately 50% of the AROs were highly enriched in the phyla Firmicutes and Bacteroidetes (Figure 6B). These results suggest that the occurrence of ARGs is influenced by specific types of gut microbes. In addition, to understand the background variation in ARGs. Base on the relative abundances of ARGs in the different species, the top 10 ARGs were enriched analysis. We found that macB, bcrA, msbA, Stre2, efrA, rpoB2, novA, evgS, tetA58, and mtrA were enriched in marmot compared to others; G418, PAR, HGM, AMK, and PAC were enriched in Alpine marmot compared to others; KAS, GENC, KAN, NEO, and STR were enriched in European rabbit compared to others (Figure 6C).

### Microbes and metabolic pathways associated with cellulose degradation

To explore the microbiota’s ability to digest carbohydrates, we compared the genes in the gut microbial metagenome of marmots with those in the KEGG and CAZy databases to screen for relevant genes. According to the KEGG analysis, 96,082 genes were classified into 3,946 KEGG ortholog groups. There were 365 genes with homologous sequences to genes encoding cellulase (EC 3.2.1.4; *n* = 292), cellulose 1,4 β-cellobiosidase (EC 3.2.1.91; *n* = 4), and β-glucosidase (EC 3.2.1.21; *n* = 69). These genes were associated with 44 bacterial and 4 fungal genera.

The cellulase genes came from many species, involving 117 bacterial, 1 fungal, and 1 archaeal species. The 117 bacteria belonged to 39 genera. The 5 most abundant bacteria were Clostridium (*n* = 15), Ruminococcus (*n* = 14), Butyrivibrio (*n* = 12), Firmicutes (*n* = 8), and Eubacterium (*n* = 8). Theβ-glucosidase genes came from 44 bacterial and 1 fungal species. The 44 bacteria belonged to 20 genera. The top 3 most abundant bacteria belonged to Clostridium (*n* = 15), Ruminococcus (*n* = 4), and Lachnospiraceae (*n* = 4). The cellulose 1,4-β-cellobiosidase genes came from 3 bacterial and 1 fungal species. The 3 bacteria belonged to Ruminiclostridium, Bacteroides, and *Coprobacillus* and the 1 fungus belonged to Saccharomyces.

**Figure 7:**
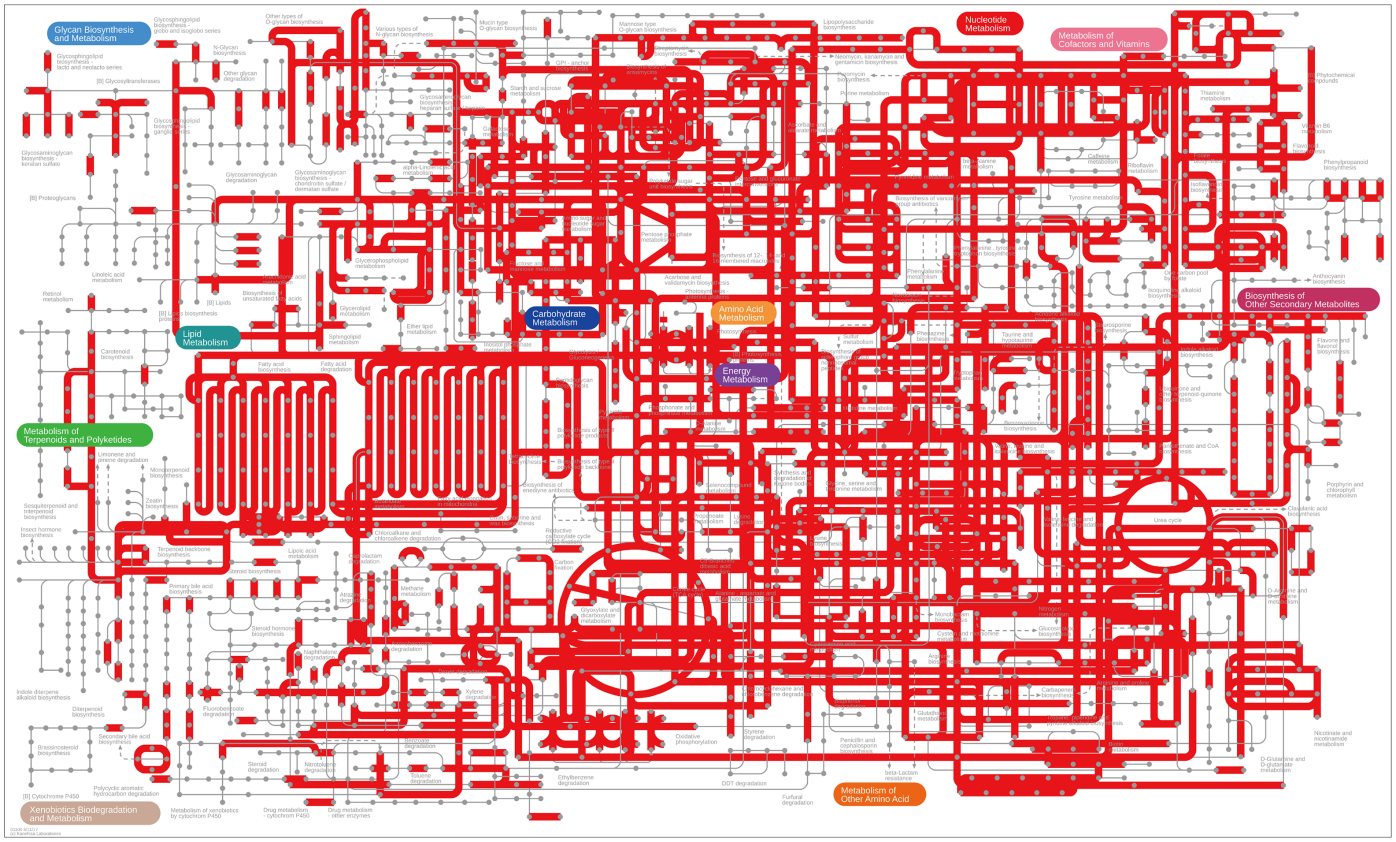
Overview of KEGG pathways of the gut microbiota, visualized using iPath.

According to the CAZy analysis, 36,989 putative GH genes were classified into 109 GH families (among the 145 GH families in the CAZy database). There were 31 GH families with >1% abundance, including GH13 (*n* = 4,169), GH2 (*n* = 2,515), GH23 (*n* = 2,508), GH3 (*n* = 2,397), GH43 (*n* = 1,975), GH73 (*n* = 1,386), GH18 (*n* = 1,185), and GH5 (*n* = 1,099; Supplementary Table S5). Additionally, there were 10 GH families with encoding cellulase, β-glucosidase, and cellulose β-1,4cellobiosidase (Supplementary Table S6). Based on metagenomics, we obtained a comprehensive overview of the KEGG metabolic pathways of the gut microbiota of marmots. These results revealed that the gut microbiota of marmots was involved in various aspects of metabolism, including the biosynthesis of energy, lipids, nucleotides, amino acids, carbohydrates, terpenoids, cofactors, polyketides, glycan, and other secondary metabolites, and the biodegradation of xenobiotics (Figure 7).

## Discussion

This study involved fecal samples from marmots from the Hulun Buir grassland of Inner Mongolia, China. The majority of metagenomic reads (80.91%) were from bacteria, 0.13% were from archaea, 0.05% were from viruses, 0.08% were from eukaryotes, and 18.83% were unclassified.

In general, the most dominant phyla in the gut microbiota of mammals are Proteobacterium, Bacteroides, and Firmicutes (Delsuc et al., 2014). Many bacteria have putative genes encoding cellulose and hemicellulose digestive enzymes. Most of the bacteria belong to Firmicutes, especially the genus Clostridium (Zhu et al., 2011). Firmicutes and Actinobacteria were lower in the gut microbiota of woodchucks compared to human, while Bacteroidetes and Fusobacterium were higher (Xiang et al., 2020). Proteobacteria, followed by Firmicutes and Bacteroidetes, were the dominant phyla in the gut microbiota of the Himalayan marmot. In our study, at the phylum level, the most abundant bacterium was Firmicutes, followed by Proteobacteria, Bacteroidetes, and Actinobacteria. At the genus level, Clostridium, Lachnospiraceae_noname, Ruminococcus, and Bacteroides were dominant. We described in detail the distinct gut bacteria of marmots, especially at the species level. The results show that the composition of the gut microbiota of marmots in the Hulun Buir grasslands had certain commonalities with that of other regions, but there were also certain differences. In addition, we found various potential cellulolytic and hemicellulolytic bacteria in the marmot gut microbiota. Bacteroidetes can help the host to degrade carbohydrates and proteins and maintains the ecological balance of intestinal microorganisms (Hooper et al., 2001; Hooper, 2004; Biely, 2012). Bacteroides increase in the gut of human eating low-fat diets in natural environments (Ley et al., 2006). Actinobacteria has been found in the gut of humans (Krogius-Kurikka et al., 2009), pandas, cheetahs (Becker et al., 2014), and mice (Murphy et al., 2010). Gastrointestinal research has shown that Actinobacteria are related to diverse environments and conditions (D’Argenio and Salvatore, 2015). Dietary habits and living conditions may cause wild marmots to acquire these bacteria.

Regarding fungal phyla Ascomycota, Basidiomycota, Blastocladiomycota, and Microsporidia were dominant. Microsporidia are obligate intracellular eukaryotic parasites (Opperman et al., 2008). Many of the fungi genera that we identified (e.g., Fusarium, Colletotrichum, Piloderma, and Rhizophagus) are associated with plants. Fusarium (Kistler, 1997) and Colletotrichum (O’Connell et al., 2012) are believed to be plant pathogens. Piloderma is a wood ectomycorrhizal fungus (Hagerberg et al., 2005). Moreover, Rhizophagus is an arbuscular mycorrhiza that can have symbiotic relationships with plants (Tisserant et al., 2013). Marmots may be exposed to these fungi *via* their diet (grasses, stems, leaves, and many other foods), water, and the environment. Until now, there were limitations regarding the understanding of the diversity and role of fungi in the gut of marmots. In this study, we identified species-level fungi using whole metagenomic shotgun sequencing, comprehensively identifying the gut fungi of marmots. In addition to bacteria, fungi play a significant role in host health and intestinal diseases (Huffnagle and Noverr, 2013; Sokol et al., 2017). Likewise, in addition to bacteria, fungi have genes encoding enzymes that digest starch, cellulose, and hemicellulose in the guts of marmots. They can aid in the digestion of substances in grasses, stems, and leaves (Zhu et al., 2011; Yang et al., 2018). We found that there were anaerobic fungi in the gut of marmots. Anaerobic fungi can produce enzymes to effectively hydrolyze cellulose and hemicellulose, which are usually present in the digestive tracts of ruminants and monogastric herbivores (Ljungdahl, 2008; Haitjema et al., 2014). For herbivores, hydrolysis of carbohydrates in plant cell walls by the gut microbiota is key for food digestion (Hooper and Macpherson, 2010; Wang et al., 2013).

We identified 233 species of virus from 5 classified orders, 22 classified families, and 53 classified genera. The most abundant classified families were Myoviridae, Siphoviridae, Phycodnaviridae, Podoviridae, Herpesviridae, Caudovirales, and Retroviridae. We found many bacteriophages in the guts of marmots, including the order Caurovirina. Research has shown that Caudovirales bacteriophages are present in the guts of patients with inflammatory bowel disease (Norman et al., 2015). Bacteriophage diversity is influenced by the environment (Lusiak-Szelachowska et al., 2017). Bacteriophages can cause gut dysbiosis, altering the ratio of commensal-to-pathogenic bacteria (Mills et al., 2013). Bacteriophages are seen as regulators of bacterial populations in the gut, influencing bacterial diversity and metabolism by lysing bacteria and controlling bacterial populations, thereby having a major impact on host health (Brown-Jaque et al., 2016; Lusiak-Szelachowska et al., 2017). We support the idea that bacteriophages with bactericidal activity may be used to treat infections in marmots in the future, as a replacement for or a supplement to antibiotics.

Archaea, first found in extreme environments, are now known to be ubiquitous (Woese et al., 1990). In the past decades, key achievements in archaea research have included the discovery of anaerobic methane oxidation (Boetius et al., 2000), thaumarchaeal ammonia oxidation (Leininger et al., 2006), the seventh order of methanogens (Paul et al., 2012; Iino et al., 2013), and the discovery of the phylum Bathyarchaeota (a non-euryarchaeal lineage with methanogenic properties (Evans et al., 2015)) and the evolutionarily important phylum Lokiarchaeota (Spang et al., 2015), to name just a few. These findings indicate the importance of archaea, and they reveal novel traits beyond archaeal extremophily and supposed “primitiveness.” Methanoarchaea strains and some archaeal strains with low immunogenicity are considered to be typical symbiotic microorganisms in the gut, while some strains with high immunogenicity are associated with the development of inflammatory gut conditions in humans (Bang et al., 2014; Blais Lecours et al., 2014; Borrel et al., 2017). As for positive effects on human health, Methanoarchaea strains are currently being considered as potential probiotics to manage metabolic disorders associated with trimethylamine produced by gut bacteria (Zhernakova et al., 2016; Borrel et al., 2017). Furthermore, a study reported on the use of unusual lipid fractions of several archaeal strains as prospective adjuvants (Krishnan and Sprott, 2008). In the current study, the most abundant archaeal genera were *Methanosarcina*, *Thermococcus*, *Methanococcus*, *Methanobrevibacter*, *Methanobacterium*, *Methanocaldococcus*, *Natrinema*, *Methanothermobacter*, *Methanolobus*, and *Methanocella*. The results revealed the diversity of the marmot gut archaea and the potential impacts on host.

Host species and environmental factors (such as geography and diet) are important factors in the formation of gut microbiota diversity (Smits et al., 2017; Fu et al., 2021). We found that the gut microbiota of the three herbivores, i.e., marmot, Alpine marmot, and European rabbit were richest in Firmicutes, which helps hosts to obtain more energy from food (Chevalier et al., 2015). We found 52 different microorganisms using LEfse analysis, which were classified into Firmicutes, Bacteroidetes, Planctomycetes, Verrucomicrobia, proteobacteria, Tenericutes, Actinobacteria, Cyanobacteria, and Elusimicrobia. Verrucomicrobia were discovered to oxidize methane and used methane as unique source of carbon and energy (Dunfield et al., 2007). Planctomycetes possessed antibiotic resistance and the ability to use N-acetylglucosamine as a carbon substrate (Schlesner, 1994). They could help the host to obtain nutrition and energy by acting on different substrates. We found that Planctomycetes were significantly enriched in the marmots, and Verrucomicrobia were significantly enriched in the European rabbis, which may be attributed to the species and dietary habits differences of the host. Actinobacteria produced mycothiol (MSH; AcCys-GlcN-Ins) with the functions of glutathione. MSH played a key role in the detoxification of alkylating agents, reactive oxygen and nitrogen species, and antibiotics (Newton et al., 2008). Compared with other two wild animals, we found Tenericutes, Actinobacteria, Cyanobacteria, and Elusimicrobia were significantly enriched in the Alpine marmots. Alpine marmot’s diet is diverse, involving grass, grain, insects, spiders and worms. Dietary habits may cause wild Alpine marmots to acquire these bacteria. However, the impact of the environment is reduced by the specific diet of the host (Knowles et al., 2019). When different host species share a habit/diet, despite the host species being evolutionarily distant, gut microbiota communities often converge (Lutz et al., 2019; Perofsky et al., 2019). We found that the BC_ij_ of Alpine marmots–European rabbits was 0.28. Alpine marmots and European rabbits share a habitat, which explains the convergence of the gut microbiota composition and structure. In addition, regional differences that lead to dietary changes are ubiquitous among wild animals, resulting in differences in the composition and structure of intestinal microbial communities (Li et al., 2018); we found that the BC_ij_ of marmots–Alpine marmots, whose living environments do not intersect, was 0.93 and the gut microbiota of the former is far richer than that of the latter despite them being the same host species. The differences in the living environment may be the reason for the variation of the composition and structure of their gut microbiota community. The species and living environment of hosts can regulate the gut microbiota diversity from different aspects (Youngblut et al., 2019). We found that the BC_ij_ of marmots–European rabbits was 0.96. Although marmots and European rabbits are both herbivores, they are different host species and live in different environments, resulting in very different gut microbiota composition and structure. In conclusion, our results showed that the composition and function of the gut microbiota of wild herbivores were shaped by the host species and environment. In addition, it was suggested that the gut microbiota structure of wild herbivores was closely related to the external environment while being regulated by the internal environment.

Our functional analysis indicated that the gut microbiota in marmots exhibited high metabolic activity. The gut microbiota was involved in various aspects of metabolism, including the biosynthesis of energy, lipids, nucleotides, amino acids, carbohydrates, terpenoids, cofactors, polyketides, glycan, and other secondary metabolites, and the biodegradation of xenobiotics. Furthermore, GO and KEGG enrichment analyzes of the five metagenomes revealed their functional diversity. In addition, Hulun Buir City in Inner Mongolia, China, has world-famous natural pastures and vast agricultural arable land, and agriculture and animal husbandry are key local industries. For the prevention and treatment of disease in the animal husbandry industry, various types of antibiotics were used, including macrolide, tetracycline, mupirocin, phenol, and aminoglycoside. Similarly, for the prevention and treatment of crop diseases and pests, antibiotics/fungicides were used, including *Bacillus subtilis*, triazole fungicides, methoxyacrylate, and polyantinomycin.^5^ We identified a range of ARG categories, which were related to the antibiotics used for prevention and treatment in the local agriculture and animal husbandry industries. We found that macB, bcrA, msbA, Stre2, efrA, rpoB2, novA, evgS, tetA58, and mtrA were enriched in marmot compared to other species. The most abundant ARG in marmots was macB, followed by bcrA and msbA. The periplasmic region of macB (a noncanonic ABC transporter) in Gram-negative bacteria is responsible for the outflow of macrolide antibiotics and the secretion of heat-stable enterotoxin II (Modali and Zgurskaya, 2011). BcrA and BcrB are subunits of ATP-binding cassette (ABC) antibiotic efflux pumps that play a major role in mediating bacitracin resistance in Gram-positive bacteria (Tremblay et al., 2011). MsbA is a typical multidrug-resistant protein in the ABC transporter family (Campbell et al., 2003). Furthermore, the majority of AROs in marmots originated from the dominant phyla Firmicutes and Bacteroidetes. In addition, we found that G418, PAR, HGM, AMK, and PAC were enriched in Alpine marmot compared to other species; KAS, GENC, KAN, NEO, and STR were enriched in European rabbit compared to other species. The intestinal microbes in wild animals synthesize ARGs (Carter et al., 2018) and the animals can also obtain ARGs from the external environment (Li et al., 2021). Due to the long-term abuse of antibiotics in the animal husbandry and aquaculture industries, resistant strains harboring ARGs are found in the intestines of farm animals and can be excreted in feces. After rain, the surface runoff and atmospheric diffusion leads to these resistant strains entering the environment where they can be ingested by animals (Li et al., 2021). ARGs can be transferred *via* horizontal plasmid exchange from bacteria in the environment to human or livestock microbiota (Lim et al., 2020). Some ARGs are enriched in one species compared to others, they are likely due to environmental exposure to antibiotics. We suspected that ARGs in marmots, Alpine marmots, and European rabbits may originate from the above pathways. We believe that it is necessary to reduce (e.g., reducing the use of unnecessary antibiotics) the environmental pollution risk related to ARGs in animal feces, preventing dispersal to human and animals.

Cellulose is the main component of the cell walls of plants (Naoto and Mitsuo, 2014). Similarly, hemicelluloses are found in the cell walls of plants that have β (1 → 4)-linked backbones with an equatorial configuration (Scheller and Ulvskov, 2010). The β-(1 → 3,1 → 4)-glucans in grass cell walls and some of the arabinoxylans in cereal endosperm are hemicelluloses (Scheller and Ulvskov, 2010). The nutrients of herbivores mainly come from cellulose and hemicellulose in plants. As the nutrient density of forage is very low, herbivores need to ingest a large amount of forage to supply their own nutrient needs. To digest food better, herbivores not only need to chew food repeatedly, but also rely on the gut microbiota to help with fermentation and digestion. To understand whether marmots have gut microbiota that is adapted to their fiber-rich diet, we conducted a structural analysis of the marmot gut microbiota based on genes in the metagenome. In the gut of marmots, genes encoding cellulose catabolic enzymes were found in bacteria, archaea, and fungi; the number of genes from bacteria was higher than that from fungi and archaea. Among the genes encoding cellulose catabolic enzymes, the number of cellulase genes were the largest, followed by β-glucosidase and cellulose 1,4 β-cellobiosidase. Therefore, the bacteria, fungi and archaea in the gut of marmots may play a key role in cellulose metabolism. In addition, we found many GH genes in the metagenome, indicating that the marmot gut exhibited carbohydrate metabolic activity. The four most abundant GH families (from most to least abundant) were GH13, GH2, GH23, and GH3. The GH13 family contains many enzymes active against alpha-glucan, possibly related to starch hydrolysis (Peng et al., 2011). The GH23 family contains lysozymes and soluble lytic transglycosylases (Tufariello et al., 2006; Scheurwater et al., 2008). In the GH3 family, the most abundant enzymes that we found exhibited the degradation activity of cellulose and hemicellulose β-glucosidase, followed by β-N-acetylglucosaminidases (Macdonald et al., 2015). Notably, GH1 and GH2 families also have cellulose and hemicellulose degradation activities, including β-glucosidase, β-galactosidase, β-mannosidase and β-glucuronidase, and glucan 1,4-β-glucosidase (Kuntothom et al., 2009; Heins et al., 2014; Talens-Perales et al., 2016). To sum up, the gut microbiota of marmots not only harbors members of a diverse range of GH families, but can also degrade cellulose and hemicellulose.

There are several limitations in this study. First, the sample size of this study was relatively small, which may reduce the accuracy of the results. In addition, as the fecal samples used in this study were collected from wild animals, physiological variables of the marmots were not assessed. Finally, we did not focus on collecting fecal samples from diseased marmots, but instead collected fecal samples from the general population of marmots, for background investigation of the gut microbiota. In the future, pathogenic samples should be obtained for further research.

## Conclusion

Using metagenomic sequencing, we comprehensively described microbiota structure, in the gut of marmots. We found 5,891 bacteria, 236 fungi, 233 viruses, and 217 archaea. Moreover, we found 36,989 GH genes in the marmot gut microbiota, with 365 genes with homologous sequences to genes encoding β-glucosidase, cellulase, and cellulose β-1,4-cellobiosidase. This indicates that the gut microbiota of marmots can hydrolyze cellulose, hemicellulose and starch in food, thus increasing the digestibility of the food. We found that the structure of the gut microbiota of marmots was shaped by the host species and environment. We also investigated the ARGs and found that ARGs such as macB, bcrA, and msbA were abundant. In conclusion, the results showed that the intestinal microbes of marmots had population diversity and functional diversity, which provided a basis for further research on the regulatory effects of the gut microbiota on the host.

## Data availability statement

The data presented in the study are deposited in the NCBI Sequence Read Archive (SRA) repository under BioProject accession number PRJNA946794.

## Author contributions

CC and BW conceived the idea. SC collected the samples. CC performed the experiments and the statistical analyzes, and wrote the first draft of the manuscript. BW contributed substantially to revisions. All authors contributed to the article and approved the submitted version.

## Funding

This work was supported by the National Natural Science Foundation of China (grant number 82120387); Hainan Provincial Natural Science Foundation of China (grant number 822QN318); Pathogenic spectrum, diagnosis and treatment of tick-borne infectious diseases in eastern of Inner Mongolia (grant number 2018015); Youth Cultivation Fundation of Hainan Medical University (grant number HYPY201923).

## Conflict of interest

The authors declare that the research was conducted in the absence of any commercial or financial relationships that could be construed as a potential conflict of interest.

## Publisher’s note

All claims expressed in this article are solely those of the authors and do not necessarily represent those of their affiliated organizations, or those of the publisher, the editors and the reviewers. Any product that may be evaluated in this article, or claim that may be made by its manufacturer, is not guaranteed or endorsed by the publisher.

## Footnotes

^1^ http://i.cs.hku.hk/~alse/hkubrg/projects/idba_ud/

^2^ http://huttenhower.sph.harvard.edu/galaxy/

^3^ http://circos.ca/

^4^ http://leb.snu.ac.kr/amdb

^5^ http://nmj.hlbe.gov.cn
