# Supplementary material for: A glance at the gut microbiota and the functional roles of the microbes based on marmot fecal samples
Source: Front Microbiol. 2023 Apr 14;14:1035944. doi: 10.3389/fmicb.2023.1035944 (PMC10140447; doi:10.3389/fmicb.2023.1035944)
Supplement: Supplementary file 1 [file Table_1.docx]

**Table S1 Statistical table of effective data for metagenomic sequencing**

| **Sample** | **Raw Data** | | **Clean Data** | | **Clean%** | **Q20%** | **Q30%** | **GC%** | **Host genome rate%** |
| --- | --- | --- | --- | --- | --- | --- | --- | --- | --- |
|  | **Read** | **Base** | **Read** | **Base** |  |  |  |  |  |
| M1 | 44162538 | 6.62G | 40528598 | 5.68G | 91.77 | 97.75 | 93.26 | 46.52 | 0.20% |
|  |  |  |  |  |  |  |  |  |  |
| M2 | 41461730 | 6.22G | 39778060 | 5.64G | 95.94 | 97.16 | 92.38 | 44.59 | 0.35% |
|  |  |  |  |  |  |  |  |  |  |
| M3 | 59279598 | 8.89G | 58190038 | 8.48G | 98.16 | 97.25 | 92.54 | 44.57 | 0.28% |
|  |  |  |  |  |  |  |  |  |  |
| M4 | 57657872 | 8.65G | 56608338 | 8.26G | 98.18 | 97.44 | 92.9 | 45.1 | 0.21% |
|  |  |  |  |  |  |  |  |  |  |
| M5 | 42623370 | 6.39G | 41700830 | 6.08G | 97.84 | 97.66 | 93.05 | 44.52 | 0.20% |
| Total | 245185108 | 36.77G | 236805864 | 34.14G |  |  |  |  |  |
|  |  |  |  |  |  |  |  |  |  |
| Average | 49037022 | 7.35G | 47361173 | 6.82G | 96.38 | 97.45 | 92.83 | 45.06 | 0.24% |
|  |  |  |  |  |  |  |  |  |  |

Raw Data/Read: Sequencing raw data, with four lines as one unit, count the number of sequenced sequences after splicing of each file; Raw Data/Base: The number of sequenced sequences is multiplied by the length of the sequenced sequence, and expressed in unit G; Valid Data/Read: After preprocessing, count the number of sequenced sequences after splicing in each file in units of four lines; Valid Data/Base: After preprocessing, multiply the number of sequenced sequences by the length of the sequenced sequence, and use Unit G means; Valid Ratio% (clean%) is the ratio of valid data (Valid) to original data (Raw), expressed as a percentage; Q20% is the proportion of data with data quality ≥Q20 in valid data; Q30% is valid data with data quality ≥Q30 Data ratio; GC% data GC content in valid data.
