# Supplementary material for: A glance at the gut microbiota and the functional roles of the microbes based on marmot fecal samples
Source: Front Microbiol. 2023 Apr 14;14:1035944. doi: 10.3389/fmicb.2023.1035944 (PMC10140447; doi:10.3389/fmicb.2023.1035944)
Supplement: Supplementary file 2 [file Table_2.docx]

**Table S2 Top 10 bacteria, fungi, archaea, and virus in marmot's gut at genus level**

| **Genus** | **Relative abundance** |
| --- | --- |
| k_Bacteria/g__Clostridium | 28.77% |
| k_Bacteria/g__Vibrio | 18.83% |
| k_Bacteria/g__Ruminococcus | 7.08% |
| k_Bacteria/g__Lachnospiraceae_noname | 4.12% |
| k_Bacteria/g__Bacteroides | 3.06 |
| k_Bacteria/g__Prevotella | 1.83% |
| k_Bacteria/g__Firmicutes_noname | 1.47% |
| k_Bacteria/g__Bacteria_noname | 1.41% |
| k_Bacteria/g__Eubacterium | 1.36% |
| k_Bacteria/g__Paenibacillus | 1.31% |
| k_Fungi/g__Saccharomyces | 3.67% |
| k_Fungi/g__Candida | 3.41% |
| k_Fungi/g__Penicillium | 2.62% |
| k_Fungi/g__Exophiala | 2.48% |
| k_Fungi/g__Pichia | 2.44% |
| k_Fungi/g__Aspergillus | 2.25% |
| k_Fungi/g__Rhodotorula | 2.23% |
| k_Fungi/g__Ustilago | 2.22% |
| k_Fungi/g__Fusarium | 2.07% |
| k_Fungi/g__Yamadazyma | 1.94% |
| k_Fungi/g__Metarhizium | 1.76% |
| k_Archaea/g__Methanosarcina | 6.53% |
| k_Archaea/g__Thermococcus | 3.27% |
| k_Archaea/g__Methanococcus | 3.24% |
| k_Archaea/g__Methanobrevibacter | 2.85% |
| k_Archaea/g__Methanobacterium | 2.46% |
| k_Archaea/g__Methanocaldococcus | 2.36% |
| k_Archaea/g__Natrinema | 2.34% |
| k_Archaea/g__Methanothermobacter | 2.10% |
| k_Archaea/g__Methanolobus | 1.97% |
| k_Archaea/g__Methanocella | 1.77% |
| k_Virus/g__Caudovirales_noname | 7.99% |
| k_Virus/ g__Alphalipothrixvirus | 6.64% |
| k_Virus/ g__Phikzlikevirus | 5.09% |
| k_Virus/ g__T5likevirus | 4.91% |
| k_Virus/ g__Marseilleviridae_noname | 4.09% |
| k_Virus/ g__Simplexvirus | 3.44% |
| k_Virus/ g__Scutavirus | 3.29% |
| k_Virus/ g__Intracisternal_A-particles | 2.94% |
| k_Virus/ g__Phietalikevirus | 2.65% |
| k_Virus/ g__Orthopoxvirus | 2.62% |
