# Supplementary material for: A glance at the gut microbiota and the functional roles of the microbes based on marmot fecal samples
Source: Front Microbiol. 2023 Apr 14;14:1035944. doi: 10.3389/fmicb.2023.1035944 (PMC10140447; doi:10.3389/fmicb.2023.1035944)
Supplement: Supplementary file 3 [file Table_3.docx]

**Table S3 Top 10 bacteria, virus, fungi, and archaea in marmot's gut at species level**

| **Species** | **Relative abundance** |
| --- | --- |
| k_Bacteria/s_Clostridium_sp_CAG:798 | 28.43% |
| k_Bacteria/s_Vibrio_nigripulchritudo | 26.95% |
| k_Bacteria/s_Ruminococcus_albus | 9.41% |
| k_Bacteria/s_Clostridium_sp_CAG:508 | 5.44% |
| k_Bacteria/s_Lachnospiraceae_bacterium_CAG:215 | 3.63% |
| k_Bacteria/s_Faecalibacterium_prausnitzii | 2.09% |
| k_Bacteria/s_Clostridium_sp_CAG:465 | 1.61% |
| k_Bacteria/ s_Desulfosporosinus_orientis | 1.56% |
| k_Bacteria/s_Pseudoflavonifractor_capillosus | 1.26% |
| k_Bacteria/s_Legionella_pneumophila | 1.21% |
| k_Virus/s__Acanthocystis_turfacea_Chlorella_virus_NTS-1 | 12.54% |
| k_Virus/s__Nitrincola_phage_1M3-16 | 6.83% |
| k_Virus/s__Vibrio_phage_pVp-1 | 4.73% |
| k_Virus/s__Mouse_Intracisternal_A-particle | 4.71% |
| k_Virus/ s__Streptococcus_phage_phiD12 | 4.44% |
| k_Virus/s__Bacillus_phage_Moonbeam | 4.06% |
| k_Virus/s__Staphylococcus_phage_StB12 | 2.94% |
| k_Virus/s__Clostridium_phage_c-st | 2.68% |
| k_Virus/ s__Klebsiella_phage_JD001 | 2.64% |
| k_Virus/s__Melbournevirus | 2.58% |
| k_Fungi/s__Saccharomyces_kudriavzevii | 7.36% |
| k_Fungi/s__Pichia_kudriavzevii | 7.02% |
| k_Fungi/s__Rhodotorula_marina | 6.14% |
| k_Fungi/s__Ustilago_maydis | 5.83% |
| k_Fungi/ s__Blastocladiella_emersonii | 5.06% |
| k_Fungi/s__Rasamsonia_emersonii | 4.56% |
| k_Fungi/ s__Candida_albicans | 4.34% |
| k_Fungi/s__Candida_tenuis | 4.20% |
| k_Fungi/ s__Gymnopus_luxurians | 3.45% |
| k_Fungi/ s__Mixia_osmundae | 2.96% |
| k_Archaea/s__uncultured_archaeon_GZfos23H9 | 7.36% |
| k_Archaea/s__Methanosarcina_acetivorans | 6.45% |
| k_Archaea/s__Methanocella_arvoryzae | 5.12% |
| k_Archaea/s__Thaumarchaeota_archaeon_SAT1 | 5.02% |
| k_Archaea/s__Methanolacinia_petrolearia | 4.81% |
| k_Archaea/s__Methanolobus_tindarius | 4.74% |
| k_Archaea/ s__euryarchaeote_SCGC_AAA261-G15 | 4.00% |
| k_Archaea/s__Thermococcus_sp._ES1 | 3.56% |
| k_Archaea/s__Methanosarcina_horonobensis | 3.23% |
| k_Archaea/s__Methanobacterium_sp._Maddingley_MBC34 | 3.20% |
