# Supplementary material for: A glance at the gut microbiota and the functional roles of the microbes based on marmot fecal samples
Source: Front Microbiol. 2023 Apr 14;14:1035944. doi: 10.3389/fmicb.2023.1035944 (PMC10140447; doi:10.3389/fmicb.2023.1035944)
Supplement: Supplementary file 4 [file Table_4.docx]

**Table S4 Functional annotation of genes in marmot's gut microbiota**

| **Functional annotation** | **Percent of Genes** |
| --- | --- |
| KEGG level1 |  |
| Metabolism | 59.82% |
| Genetic Information Processing | 16.59% |
| Environmental Information Processing | 14.99% |
| Cellular Procrsses | 3.01% |
| Other | 5.59% |
|  |  |
| GO annotation |  |
| Molecular Function | 26.0% |
| Biological Process | 24.5% |
| Cellular Component | 12.5% |
| NA | 37.0% |
