# Supplementary material for: A glance at the gut microbiota and the functional roles of the microbes based on marmot fecal samples
Source: Front Microbiol. 2023 Apr 14;14:1035944. doi: 10.3389/fmicb.2023.1035944 (PMC10140447; doi:10.3389/fmicb.2023.1035944)
Supplement: Supplementary file 5 [file Table_5.docx]

**Table S5 GH family more than 1% in marmots**

| **GH family** | **Numbers of gene** | **Percentage** |
| --- | --- | --- |
| GH13 | 4169 | 11.28% |
| GH2 | 2515 | 6.80% |
| GH23 | 2508 | 6.78% |
| GH3 | 2397 | 6.48% |
| GH43 | 1975 | 5.34% |
| GH73 | 1386 | 3.75% |
| GH18 | 1185 | 3.20% |
| GH5 | 1099 | 2.97% |
| GH25 | 914 | 2.47% |
| GH28 | 872 | 2.36% |
| GH39 | 872 | 2.36% |
| GH94 | 833 | 2.25% |
| GH78 | 777 | 2.10% |
| GH31 | 711 | 1.92% |
| GH53 | 699 | 1.89% |
| GH32 | 658 | 1.78% |
| GH10 | 564 | 1.52% |
| GH1 | 558 | 1.51% |
| GH16 | 552 | 1.49% |
| GH9 | 536 | 1.45% |
| GH26 | 510 | 1.38% |
| GH19 | 502 | 1.36% |
| GH77 | 500 | 1.35% |
| GH36 | 494 | 1.34% |
| GH95 | 456 | 1.23% |
| GH20 | 448 | 1.21% |
| GH33 | 434 | 1.17% |
| GH72 | 431 | 1.16% |
| GH105 | 419 | 1.13% |
| GH130 | 408 | 1.10% |
| GH51 | 393 | 1.06% |
| Total | 30775 | 83.19% |
