# Supplementary material for: A glance at the gut microbiota and the functional roles of the microbes based on marmot fecal samples
Source: Front Microbiol. 2023 Apr 14;14:1035944. doi: 10.3389/fmicb.2023.1035944 (PMC10140447; doi:10.3389/fmicb.2023.1035944)
Supplement: Supplementary file 6 [file Table_6.docx]

**Table S6 GH family encoding cellulose catabolic enzymes in marmot**

| **GH Family** | **Enzyme** | **Number of genes** | **Percentage (%)** |
| --- | --- | --- | --- |
| GH1 | β-glucosidase | 9 | 0.024 |
| GH3 | β-glucosidase | 60 | 0.162 |
| GH5 | Cellulase | 172 | 0.465 |
| GH6 | Cellulase, β-cellobiosiclase | 4 | 0.011 |
| GH8 | Cellulase | 5 | 0.014 |
| GH9 | Cellulase, β-cellobiosiclase | 100 | 0.270 |
| GH12 | Cellulase | 1 | 0.003 |
| GH44 | Cellulase | 18 | 0.049 |
| GH45 | Cellulase | 1 | 0.003 |
| GH48 | Cellulase | 6 | 0.016 |
| Total |  |  | 1.016 |
