# Supplementary figures and images for: A glance at the gut microbiota and the functional roles of the microbes based on marmot fecal samples
Source: Front Microbiol. 2023 Apr 14;14:1035944. doi: 10.3389/fmicb.2023.1035944 (PMC10140447; doi:10.3389/fmicb.2023.1035944)

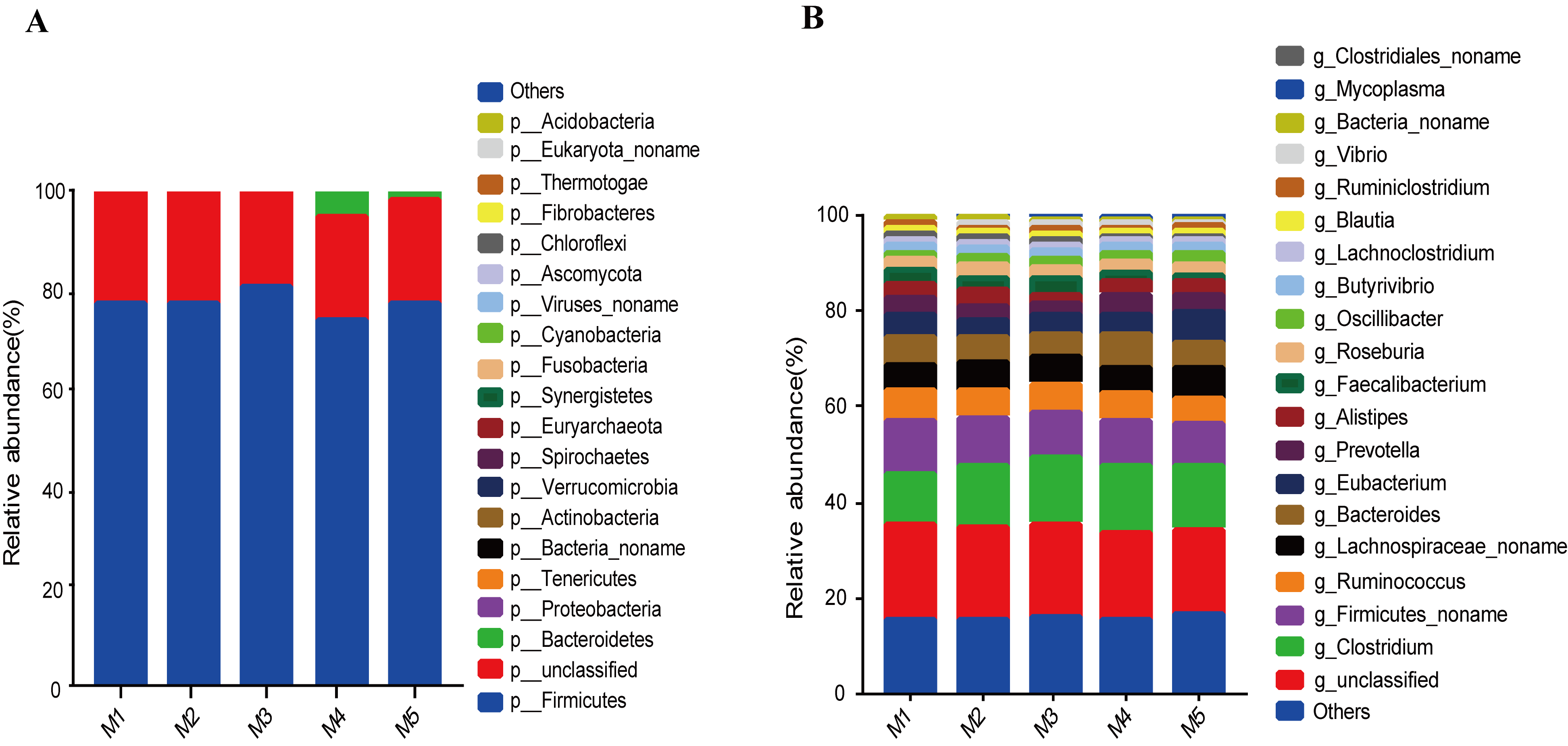

Supplement: Supplementary file 7 [file Image_1.tif]

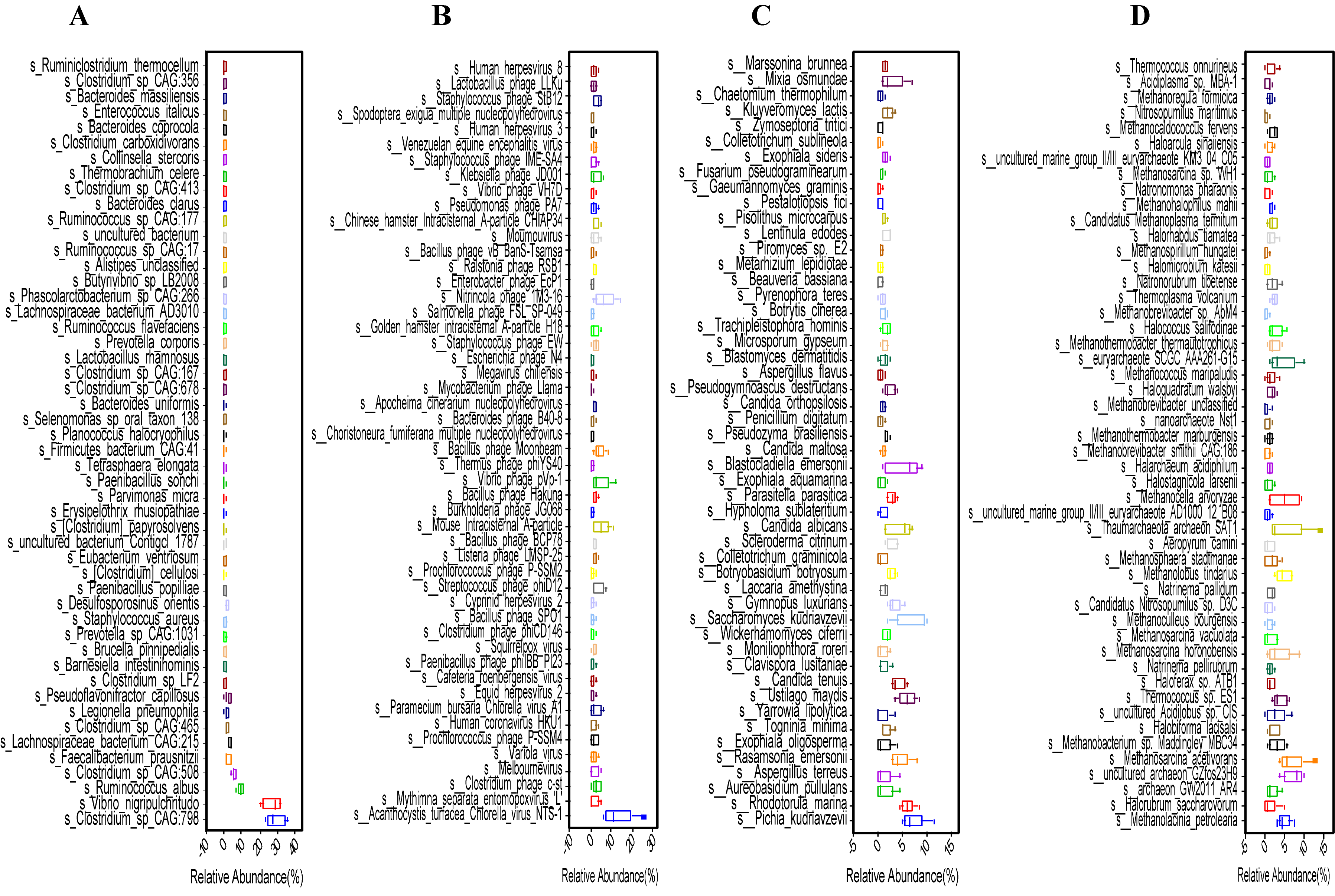

Supplement: Supplementary file 8 [file Image_2.tif]
